# Mild cognitive impairment is associated with low copy number of ribosomal genes in the genomes of elderly people

**DOI:** 10.3389/fgene.2022.967448

**Published:** 2022-09-19

**Authors:** Natalia N. Veiko, Elizaveta S. Ershova, Roman V. Veiko, Pavel E. Umriukhin, Marat V. Kurmyshev, Georg P. Kostyuk, Sergey I. Kutsev, Svetlana V. Kostyuk

**Affiliations:** ^1^ Research Centre for Medical Genetics (RCMG), Moscow, Russia; ^2^ Federal Research and Clinical Center of Intensive Care Medicine and Rehabilitology, V.A. Negovsky Research Institute of General Reanimatology, Moscow, Russia; ^3^ I.M. Sechenov First Moscow State Medical University (Sechenov University), Moscow, Russia; ^4^ P.K. Anokhin Institute of Normal Physiology, Moscow, Russia; ^5^ Mental-health Clinic No1 Named After N.A. Alexeev, Moscow, Russia

**Keywords:** ribosomal genes, rDNA, aging, rDNA CN, mild cognitive impairment

## Abstract

**Introduction**: Mild cognitive impairments (MCI) accompanying aging are associated with oxidative stress. The ability of cells to respond to stress is determined by the protein synthesis level, which depends on the ribosomes number. Ribosomal deficit was documented in MCI. The number of ribosomes depends, together with other factors, on the number of ribosomal genes copies. We hypothesized that MCI is associated with low rDNA CN in the elderly person genome.

**Materials and Methods:** rDNA CN and the telomere repeat (TR) content were determined in the DNA of peripheral blood leukocytes of 93 elderly people (61–91 years old) with MCI and 365 healthy volunteers (16–91 years old). The method of non-radioactive quantitative hybridization of DNA with biotinylated DNA probes was used for the analysis.

**Results:** In the MCI group, rDNA CN (mean 329 ± 60; median 314 copies, *n* = 93) was significantly reduced (*p* < 10^–15^) compared to controls of the same age with preserved cognitive functions (mean 412 ± 79; median 401 copies, *n* = 168) and younger (16–60 years) control group (mean 426 ± 109; median 416 copies, *n* = 197). MCI is also associated with a decrease in TR DNA content. There is no correlation between the content of rDNA and TR in DNA, however, in the group of DNA samples with rDNA CN > 540, TR content range was significantly narrowed compared to the rest of the sample.

**Conclusion:** Mild cognitive impairment is associated with low ribosomal genes copies in the elderly people genomes. A low level of rDNA CN may be one of the causes of ribosomal deficit that was documented in MCI. The potential possibilities of using the rDNA CN indicator as a prognostic marker characterizing human life expectancy are discussed.

## Introduction

Mild cognitive impairment (MCI) is defined as a transition state from the normal aging process of the brain to dementia and Alzheimer’s disease (Sanford, 2017; Jongsiriyanyong and Limpawattana, 2018). Inflammatory cytokines levels augmentation (Kinney et al., 2018; Corbo et al., 2021) and leukocyte telomere length (LTL) shortening (Liu et al., 2016; Scarabino et al., 2020) are considered to be MCI and dementia markers. It was shown that high IL-1β and IL-18 together with leukocyte LTL shortening greater than expected for person’s age, are typical signs of MCI and are detectable already at an early stage of disease (Corbo et al., 2021). Systemic oxidative stress and chronic inflammation are most probable factors of rapid LTL shortening (Blackburn et al., 2015).

The ability of cells to respond to oxidative stress is determined by the level of protein synthesis, depending on the ribosomes number (Tahmasebi et al., 2018; Baßler and Hurt, 2019; Porokhovnik and Lyapunova, 2019; Kampen et al., 2020; Narla and Ebert, 2010). The number of ribosomes depends, together with other factors, on the number of ribosomal genes copies (Larson et al., 1991; Laferte et al., 2006). Ribosomal genes (rDNA) encode rRNA, which along with ribosomal proteins forms the ribosome. Cells with a low content of rDNA in the genome in response to stress can additionally synthesize a lower number of ribosomes; as a result, the level of protein synthesis necessary for stress response will not be provided (Beĭko et al., 2005). The appearance of unrepaired damage in brain cells can lead to earlier age-related impairments of a person’s cognitive abilities.

**FIGURE 1:**
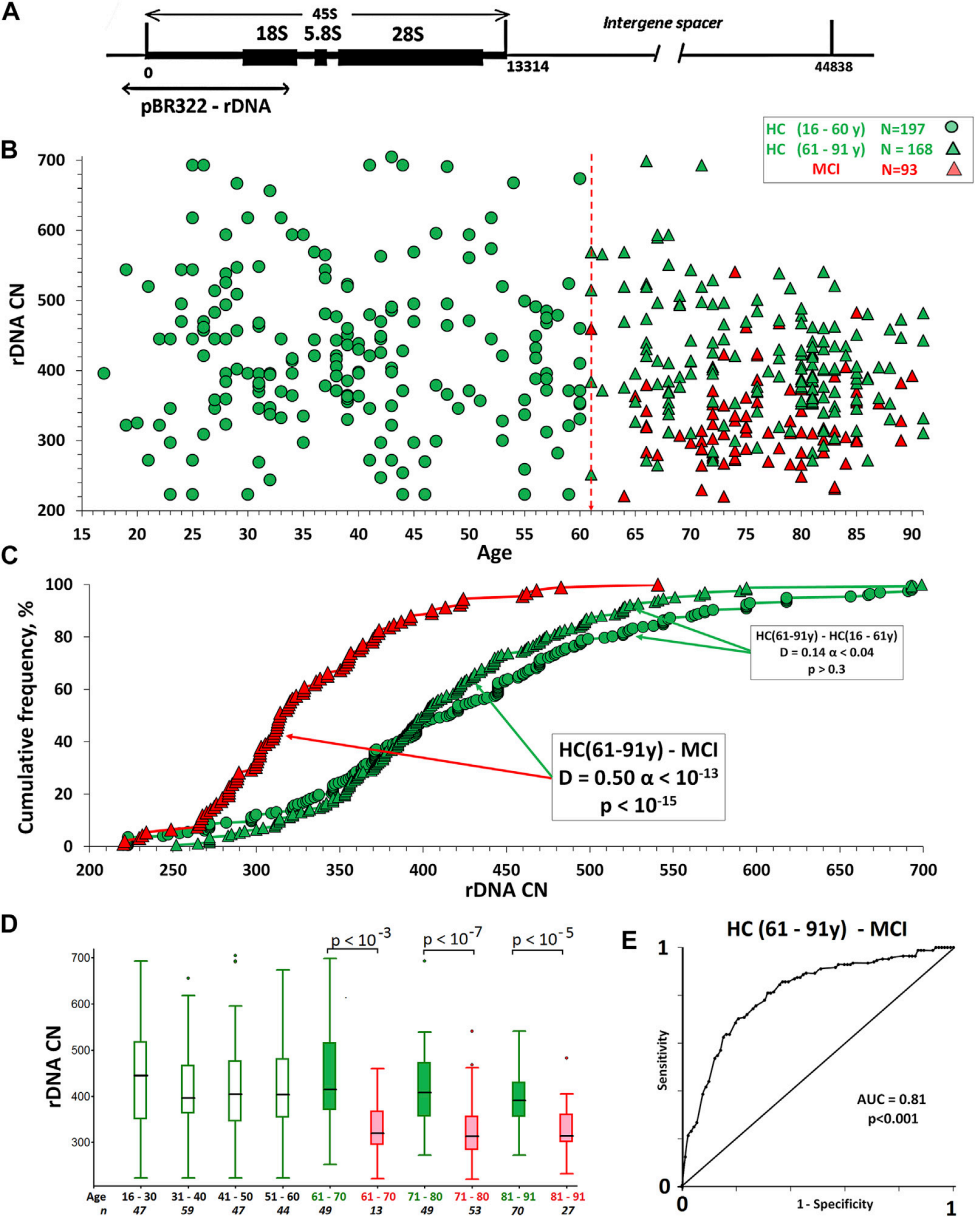
**(A)** Scheme of ribosomal repeat illustrating the DNA probe used to analyze the rDNA CN in human DNA. **(B)** The dependence of the rDNA CN in the genomes of HC groups and MCI group on age. **(C)** Cumulative distributions of DNA samples by the rDNA CN in the studied groups. The arrows indicate the distributions being compared. The data of the Kolmogorov-Smirnov (D and α) and Mann-Whitney (p) test are presented in the boxes. **(D)** A box plot showing the rDNA CN distribution for each age group. The groups were divided into smaller subgroups, about 10 years apart. **(E)** ROC curve for the HC (61–91y)/MCI groups. The area under the ROC curve (AUC) is a measure of how well a parameter can distinguish between two groups.

The human genome contains from 100 to 700 copies of tandem ribosomal repeats in a single cell (Agrawal and Ganley, 2018; Malinovskaya et al., 2018; Parks et al., 2018; Hori et al., 2021). Each repeat unit with a length of 44,838 nucleotide pairs contains a transcribed region (Figure 1A), which encodes the ribosomal RNA precursor—45S rRNA, and a non-transcribed intergenic spacer. 45S rRNA processing provides synthesis of 18, 5.8, and 28S rRNAs. Ribosomal tandem repeats are localized in the p-arms of five acrocentric chromosomes (Gonzalez and Sylvester 2001; Hori et al., 2021).

Quantitative analysis of rDNA in the genome using standard qPCR and sequencing methods presents significant difficulties (Chestkov et al., 2018; Korzeneva et al., 2016). In 2021 for the first time the number of rDNA copies (rDNA CN) was estimated by applying a new approach (“Oxford Nanopore sequencing”) to the long DNA fragments sequencing (Hori et al., 2021). Using a small sample, the authors determined that the human genome contains from 250 to 700 rDNA copies. At the same time, the authors of the present study previously proposed a method of non-radioactive quantitative hybridization, which made possible to determine the rDNA CN in the human genome (Chestkov et al., 2018; Malinovskaya et al., 2018). In the genomes of a large sample of people of different ages, the rDNA CN varied from 200 to 711, coinciding very well with the new sequencing method data. It was shown that in a sample of mentally healthy elderly people, the range of variation in the rDNA CN significantly narrows to 250–500 copies. It has been shown in cell cultures that the rDNA CN in the genome is a constant value and does not change during replicative aging, with only few cases exception when the genome contains hypermethylated rDNA copies. These copies may be lost during the aging process (Malinovskaya et al., 2018).

Since MCI is associated with oxidative stress and inflammation, it can be assumed that the cells of the human organism with MCI do not respond effectively enough to stress-inducing factors in the aging process. One of the reasons may be the low level of ribosome biogenesis, due to the small rDNA CN. Ribosomal deficit was documented in MCI (Ding et al., 2005).

We hypothesized that MCI is associated with low rDNA CN in the genome of an elderly person. To test this hypothesis, we analyzed rDNA CN in the genomes of elderly people with MCI and people with normal for their age cognitive abilities.

## Materials and methods

### Participants

The Table 1 shows demographic and clinical measures in the HC groups and MCI group.

**TABLE 1:** Demographic and clinical measures in the MCI patients and HC groups.

Index	HC(16–60y)	HC(61–91y)	MCI
N	197	168	93
Age: Range, years	16–60	61–91	61–91
Mean ± SD	35.3 ± 11.2	76.4 ± 7.7	76.5 ± 6.5
Gender (M/W)	140/57	64/104	31/62
MMSE scale: Range, points	-	26–30	24–26
Mean ± SD		28.34 ± 0.64	24.95 ± 0.87

### Control groups

DNA samples of the healthy control group (HC group, N = 365) were obtained from the collection of the Molecular biology laboratory of the Research Centre for Medical Genetics. The sample includes mentally healthy people aged 16–91 years without genetic pathology and not suffering from cognitive impairment or mental illness. In the tested sample we identified a group of people older than 60 years (N = 168), with age corresponding to the group of patients with MCI (61–91 years). These individuals were previously characterized in another study using the Mini-mental State Examination (MMSE) scale (Folstein et al., 1975).

### Patients

The study included 93 participants of the rehabilitation program “Memory Clinic” aged 61–91 years (MCI group). The results of the rehabilitation program developed at the N.A. Alekseev Psychiatric Hospital no. One were published earlier (Kurmyshev et al., 2018). The sample included 62 women (average age 73 ± 5.8 years) and 31 men (average age 79 ± 5.4 years).

Inclusion criteria: patients’ complaints about cognitive problems; identified cognitive impairments in psychometric tests using the MMSE scale (Folstein et al., 1975), cognitive dysfunction diagnosis carried out by two experts comparing the tests results; not impaired adaptation in everyday life; non-compliance with dementia criteria (Albert et al., 2011); absence of pronounced somatic pathology.

Exclusion criteria: signs of decompensation of pathology of internal organs; alcohol or psychoactive substances abuse; severe mental pathology (affective disorders, schizophrenic spectrum disorders, etc.).

Blood sampling was carried out from the cubital vein on an empty stomach in the morning between 8.00 and 8.30 in test tubes with EDTA. Blood was centrifuged (750g, 15 min, 22°C) and plasma was separated. Frozen leukocyte mass samples were stored at temperature -24 °C for a month.

The study was carried out in accordance with the latest version of the Declaration of Helsinki and was approved by the Independent Interdisciplinary Ethics Committee on Ethical Review for Clinical Studies [Protocol №4 (dated 15 March 2019)]. All participants signed an informed written consent to participate in the study after the procedures had been completely explained.

### Determination of the rDNA CN

DNA isolation from blood cells and the method of non-radioactive quantitative hybridization were described in detail in previous reports (Chestkov et al., 2018). Briefly: DNA was isolated by extraction with organic solvents after cell lysis (sodium sarcosylate and EDTA) and consequent treatment with RNase A and proteinase K. Proteinase K treatment of the lysate is critically important for more effective rDNA extraction. (Veiko et al., 2003). Extraction was carried out twice with a water-saturated (pH = 7.5) phenol solution, then with a mixture of chloroform and isoamyl alcohol. DNA from the aqueous phase was precipitated with 70% ethanol. The precipitate was washed with 75% ethanol and dissolved in water.

The success of NQH depends on the accurate DNA quantification. We perform quantification in two steps. The first one gives a rough estimation of the initial DNA amount by UV spectroscopy method. The final DNA quantification is performed fluorimetrically using the PicoGreen dsDNA quantification reagent by Molecular Probes (Invitrogen, CA, United States). For NQH, DNA solutions with the same concentration were prepared (20 ng/µL).

DNA was denatured with 0.1 M NAOH. Four dots (2 μL, 40 ng DNA in dot) were applied per each sample. Six standard samples of the genomic DNA (50 ng/ml) with a known rDNA content were applied to the same filter to plot a calibration curve for the dependence of the signal intensity on the number of rDNA copies in a particular sample. As a DNA probe, the pBR322-rDNA plasmid was used, containing a fragment of the transcribed rDNA region with a length of 5836 nucleotide pairs, cloned into the pBR322 vector at EcoRI restriction sites (Figure 1A). The cloned fragment is localized in the position from -515 to 5321 on human rDNA (GenBank access No. U13369). The empty vector pBR322 was used as a negative control. Biotin was introduced into pBR322-rDNA and pBR322 by nick translation using labeled biotin-11-dUTP.

After hybridization of DNA with a biotinylated DNA probe, biotin was detected by streptavidin conjugate with alkaline phosphatase (Sigma). A BCIP-NBT pair was used as a substrate, which, when cleaved by alkaline phosphatase, form an insoluble precipitate on the filter. The dried filter with spots was scanned. The intensity of the spots on the filter was analyzed using the Imager7.0 program (Research Centre for Medical Genetics). Calibration dependencies relating the signal intensity to the rDNA CN were published earlier (Chestkov et al., 2018). The relative standard error of the hybridization method is 5%. The average standard error of the experiment, including all procedures (DNA isolation, DNA concentration determination and hybridization method) is 11% of the measured value.

### TR content determination

For the human TR detection the following probe was used: biotin-(TTAGGG)_7_. Syntol (Moscow, Russia) performed the synthesis and biotin labeling of the oligo-probe. Calibration dependencies relating the signal intensity to the TR repeat content in DNA were published earlier (Umriukhin et al., 2022).

### Statistical analysis

The analysis of the rDNA CN and TR content in the DNA of blood cells was carried out twice in independent experiments. In one experiment, four parallel dots of the same tested sample were applied to the filter. The rDNA and TR groups were compared by the Mann-Whitney method (p). The differences were considered significant at *p* < 0.01. The distributions of DNA samples by the content of the repeats were compared by the Kolmogorov-Smirnov method (D and α). The analysis of correlations between the parameters was performed by the Spearman method (correlation coefficient Rs and probability p). The data was analyzed using the StatPlus2007 program (http://www.analystsoft.com).

MedCalc (https://www.medcalc.org/manual/roc-curves.php) creates a complete sensitivity/specificity report [Receiver Operating Characteristic (ROC) curve analysis]. Each point on the ROC curve represents a sensitivity/specificity pair corresponding to a particular decision threshold. The area under the ROC curve (AUC) reflects the parameter difference between two groups (disease/health).

## Results

### Description of the MCI group

The Table 1 shows demographic and clinical measures in the MCI group. The study included 93 participants of the rehabilitation program “Memory Clinic” (Kurmyshev et al., 2018) aged 61–91 years and 365 healthy volunteers aged 16–91 years (HC group) who did not have cognitive and memory impairments. The reason for the patient’s visit to the clinic was significant memory impairment and cognitive disorders. Cognitive impairments in MCI group patients were confirmed by psychometric testing using the MMSE scale (Folstein et al., 1975). The values of the MMSE scale index for the MCI group ranged between 24 and 26 points. The values of the MMSE scale index for the HC (61–91 y) group ranged between 26 and 30 points (Table 1).

### TR content in DNA of HC and MCI groups

In addition to psychometric testing, we conducted a biochemical test for LTL determination. It has previously been shown that the leukocyte DNA of patients with MCI contains fewer TR copies than the DNA of healthy people of similar age (Corbo et al., 2021; Scarabino et al., 2020).

**FIGURE 2:**
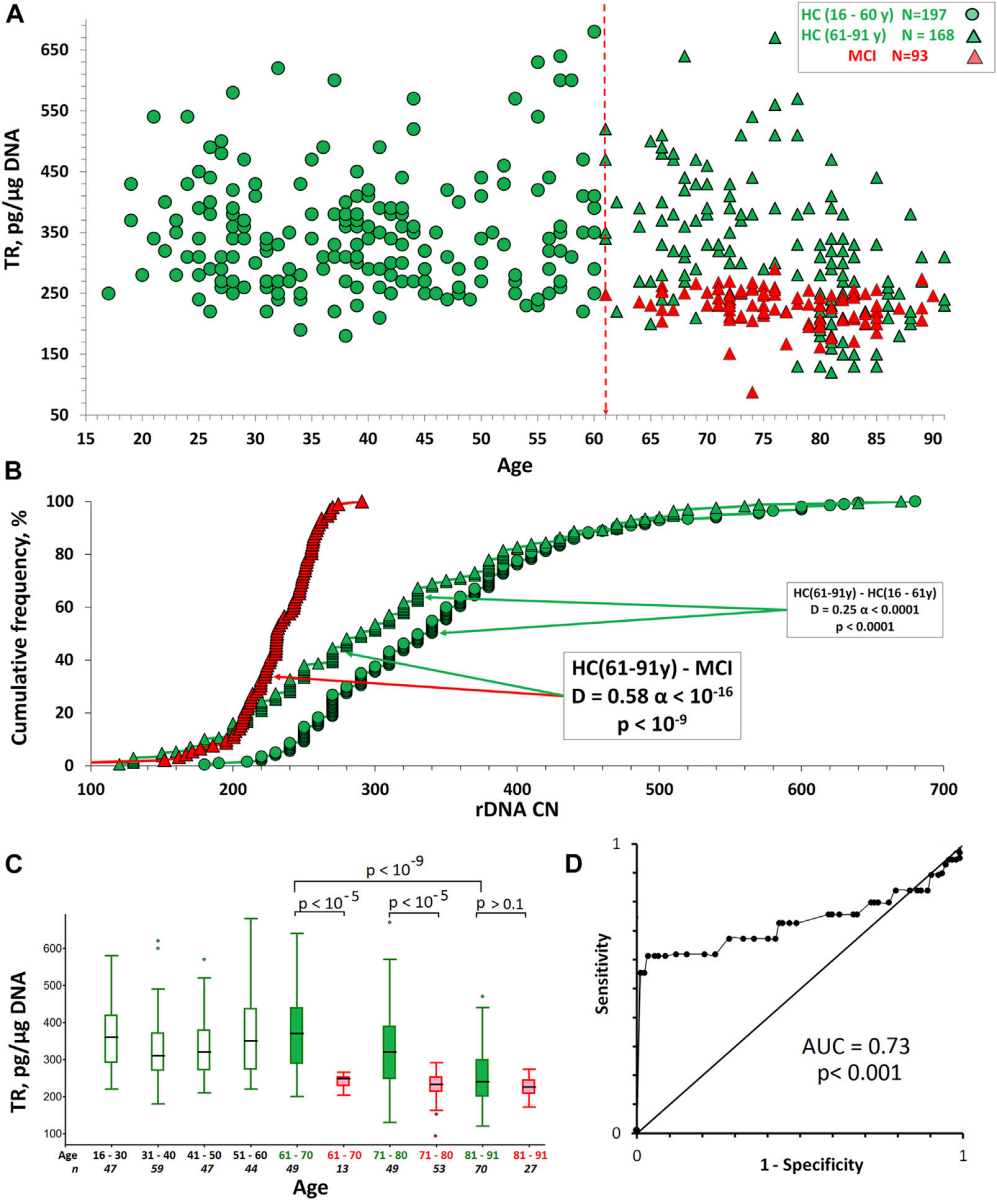
The TR content in the DNA of HC and MCI groups. **(A)** TR content dependence on human age. **(B)** Cumulative distributions of DNA samples by the TR content in the studied groups. The arrows indicate the distributions being compared. The data of the Kolmogorov-Smirnov (D and α) and Mann-Whitney (p) test are presented in the boxes. **(C)** A box plot showing the TR content distribution for each age group. The groups were divided into smaller subgroups, about 10 years apart. **(D)** ROC curve for the HC (61–91y) and MCI groups.

Figures 2A,C shows the dependence of the TR content in DNA on the age for HC and MCI groups. Table 2 illustrates descriptive statistics. Table 3 shows the correlations between the age of the people and TR content. The TR content in DNA was determined in units: pg TR/µg DNA. The maximum TR content was found in the HC (16–60 y) group. The average HC (16–60 y) group value is 348 pg TR/µg of DNA, corresponding to the average LTL, approximately 7 kb. The TR content in the DNA of MCI group was significantly reduced compared to the control group HC (61–91 y) of the same age (*p* < 10^–9^).

**TABLE 2:** Descriptive statistics data for rDNA CN and TR content in DNA.

Index	Group	N	Mean	SD	Range	Median		C_*v*_
rDNA CN	HC (16–60 y)	197	426	109	223–705	416	1	0.26
	HC (61–91 y)	168	412	79	252–699	401	0.96	0.19
	MCI (61–91 y)	93	329	60	220–541	314	0.75	0.18
TR, pg/µg DNA	HC (16–60 y)	197	348	96	180–680	340	1	0.27
	HC (61–91 y)	168	308	107	120–670	290	0.85	0.35
	MCI (61–91 y)	93	230	30	88–291	231	0.68	0.13
	HC (rDNA CN<540)	323	331	107	120–680	320	1	0.32
	HC (rDNA CN>540)	42	323	69	210–450	315	0.98	0.21
	MCI (rDNA CN<380)	78	226	31	88–274	248	0.77	0.14
	MCI (rDNA CN>380)	15	246	19	210–291	230	0.71	0.07

SD, is the standard deviation; Cv, is the coefficient of variation.

**TABLE 3:** Spearman’s rank correlations [Rs (p)] between the analyzed parameters in the HC and MCI groups.

Group	Index	rDNA CN	TR content	P (rDNA*TR)
HC (*n* = 365)	Age (16–91 y)	-0.11 (0.03)	-0.23 (<10–4)	-0.25 (<10–4)
	rDNA CN		0.03 (0.52)	0.62 (<10–4)
	TR content			0.79 (<10–4)
MCI (*n* = 93)	Age (61–91 y)	0.05 (0.66)	-0.18 (0.08)	-0.06 (0.58)
	rDNA CN		0.26 (0.012)	0.87 (<10–4)
	TR content			0.68 (<10–4)
HC (*n* = 168)	Age (61–91 y)	-0.24 (0.002)	-0.47 (<10–4)	-0.49 (<10–4)
	rDNA CN		0.07 (0.36)	0.54 (<10–4)
	TR content			0.87 (<10–4)

In the HC (16–60 y) group there is no correlation between age and TR content (Rs = 0.08, *p* = 0.28, N = 197). In the HC (61–91 y) group, there is a decrease in LTL depending on age (Rs = -0.47, *p* < 10^–4^, N = 168). In the MCI group, the negative correlation between age and LTL (Table 3) is nonsignificant (Rs = -0.18, *p* = 0.08, N = 93). The differences between the mean values of the TR content for HC and MCI groups disappear at the age of 81–91 years (Figure 2C).

Therefore, in our study LTL in MCI group was reduced compared to people without signs of MCI, which corresponds to previously obtained data (Corbo et al., 2021; Scarabino et al., 2020).

Receiver operating characteristic (ROC) curves were analyzed to predict the absence of MCI in the elderly HC (61–91 y) people (Figure 2D). ROC curves were derived by plotting the relationship between the specificity and sensitivity at various cut-off levels. The accuracy of TR content in DNA as a diagnostic tool to differentiate HC from MCI was measured using the area under the ROC curve (AUC). The AUC was 0.73, so the accuracy was considered not good.

### rDNA content in the HC and MCI groups

Figure 1B shows the dependence rDNA CN on the age in the HC groups and in the MCI group. Table 2 provides descriptive statistics. Table 3 shows the correlations between the age and rDNA CN. In the HC (16–91 y) group rDNA CN does not depend on age (Rs = -0.11, *p* > 0.01, N = 365). In the HC (61–91 y) group rDNA CN depends on the age (Rs = -0.24, *p* = 0.002, N = 168). In the HC subgroups after 70 years rDNA CN range narrowing and variation coefficient decrease from 0.25 [HC(16–70y), N = 258] to 0.15 [HC(71–91 y); N = 107] were found (Fig.1D). In the HC (81–91 y) subgroup, there are no DNA samples with very low (below 270) and very high (above 530) rDNA CN (Fig.1C,D). In the younger HC (16–61 y) group rDNA CN varies from 223 to 705 copies (Table 2). These data completely correspond to the data previously obtained on another sample (Malinovskaya et al., 2018).

DNA samples of the MCI group contain fewer rDNA copies (*p* < 10^–15^) than the HC group samples of the similar age. (Figures 1C–E and Table 2). Distributions of the DNA samples according to the rDNA CN in the two groups differ significantly (α < 10^–13^). In the group of patients with MCI, there are no samples with very high rDNA content (above 550 copies), but it contains up to 30% of samples with a low rDNA CN (less than 300). The content of such DNA samples in the HC (61–91 y) group does not exceed 5%.

The ROC curves were analyzed to predict the absence of MCI in the HC (61–91 y) group (Figure 1E). The AUC was 0.81, so the accuracy was considered to be good. The best compromise between sensitivity (64%) and specificity (85%) was achieved using a threshold of 380 rDNA CN.

### TR content dependence on rDNA CN

Thus, we found a decrease in the content of rDNA and TR in the MCI group compared with HC group. It was interesting to find out to what extent the TR content depends on the rDNA CN in the genome. Is the content of the repeats similarly reduced in the same DNA samples or changes independently?

**FIGURE 3:**
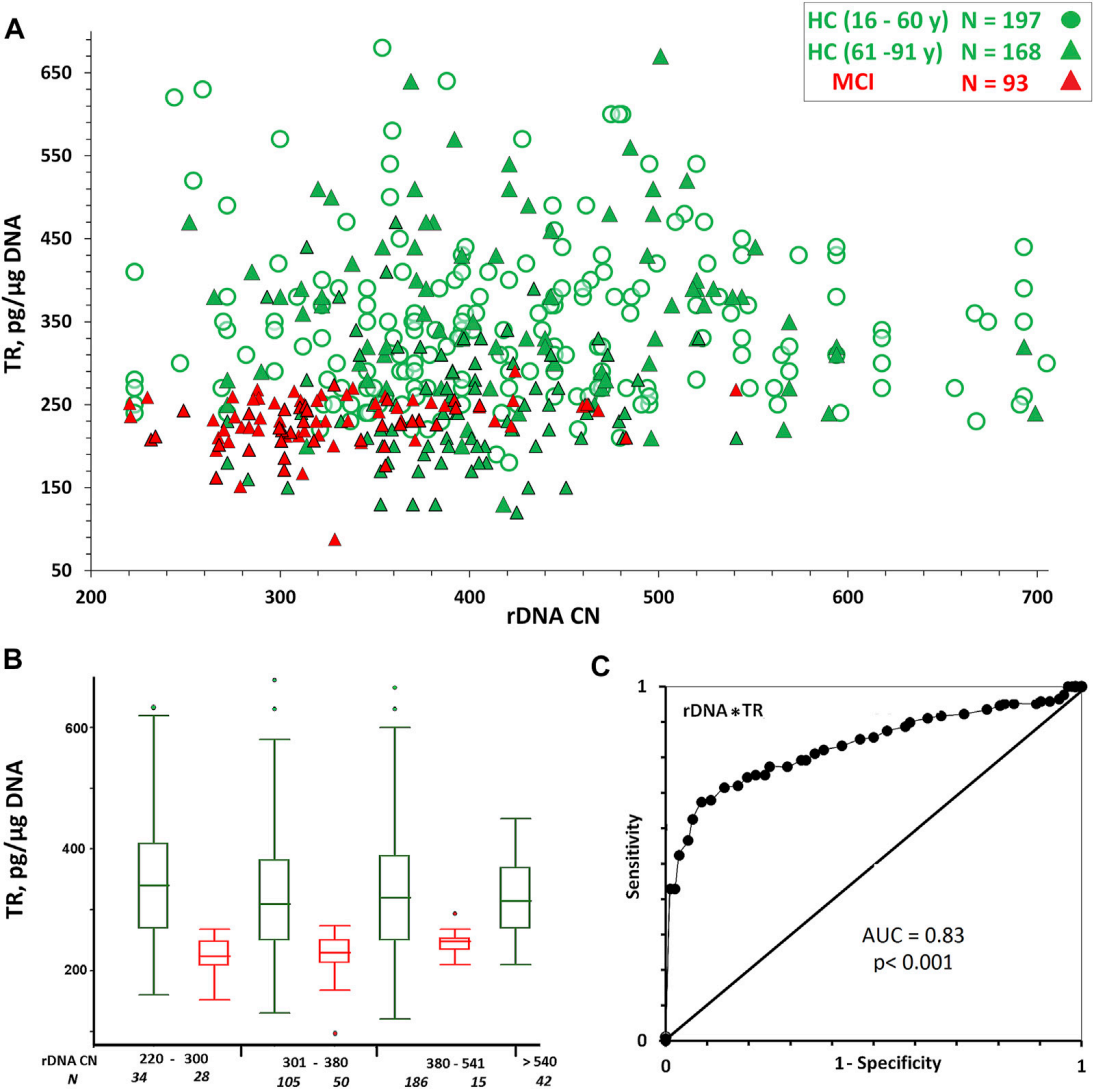
**(A)** Dependence of TR content in DNA on rDNA CN. **(B)** A box plot showing the TR content distribution for each group. The groups were divided into smaller subgroups, about 60–80 rDNA CN apart. **(C)** ROC curve for the HC (61–91y) and MCI groups. Parameters P (rDNA*TR) was analyzed.

Figure 3 and Table 3 show the TR content dependence on rDNA CN in the same DNA samples. In HC (16–60 y) and HC(61–91 y) groups the TR content in DNA is independent of rDNA CN (*p* > 0.1, Table 3). An insignificant positive correlation (Rs = 0.26, *p* = 0.012) between the content of the repeats was observed in the MCI group (Table 3).

However, it was found that the LTL range and the variation coefficient for TR content in DNA samples from the HC group with a relatively high rDNA CN (>540) were significantly reduced compared to the range and the variation coefficient in the group of samples with a lower rDNA CN (Figure 3B and Table 2).

In the HC subgroup with high rDNA CN (>540), there are no samples with very low or high TR content in DNA. A narrowing of the LTL range is also observed in the MCI subgroup with a relatively high rDNA CN for this group (>380) (Figure 3B and Table 2).

The accuracy of the index P (rDNA CN *TR content) as a diagnostic tool to separate HC (61–91y) from MCI was measured using the area under the ROC curve (Figure 3C). The AUC was 0.83 for the P index, which was higher than the value for the rDNA CN index (Figure 1E). Thus, the combined use of the two measures (rDNA CN and TR content) improves the accuracy of MCI prediction. In the MCI group, the P score is more dependent on the rDNA CN score than on the “TR content” score. In the HC (61–91 y) group, the P score is more dependent on the “TR content” score than on the rDNA CN score (Table 3).

## Discussion

In the present study, we identified for the first time the association of a relatively low rDNA CN in the genomes of elderly people with MCI manifestation (Figure 1). The question arises: did these patients have a low rDNA CN since their birth, or did the genome of the patients with MCI lose part of the rDNA copies during aging?

There are several studies of rDNA CN and aging association. The reduced rDNA CN was found to be associated with aging (Gaubatz and Cutler, 1978; Strehler et al., 1979; Das et al., 1986; Zafiropoulos et al., 2005; Ren et al., 2017). An increase in the rDNA CN was found in the brains of old people with signs of dementia (Hallgren et al., 2014; Pietrzak et al., 2011). However, some studies did not confirm observations of aging-associated rDNA CN changes (Peterson et al., 1984; Halle et al., 1997; Machwe et al., 2000). RDNA CN in the human cerebral cortex revealed no effects of normal aging on the rDNA CN (Hallgren et al., 2014). It should be noted that small numbers of samples were tested in the cited studies.

Our analysis on large sample of people of various ages (N = 651) (Malinovskaya et al., 2018) showed that in the elderly group, the mean rDNA CN is the same, but the range is narrower compared with the younger subjects (see also Figure 1D). During replicative senescence, the human fibroblast genome loses only hypermethylated rDNA copies. In the elderly group, DNA samples that contain hypermethylated rDNA copies are less common (Malinovskaya et al., 2018).

We believe that the inconsistency of the data on the association of rDNA and aging is due to two reasons. First, the groups analyzed by the authors could contain different numbers of DNA samples with hypermethylated rDNA copies that are lost during aging. Hypermethylated copies of rDNA comprise approximately 10% of human genomes. (Lyapunova and Veiko, 2010). Secondly, the analysis of rDNA CN depends on the method used. At present, the main method for the quantification of various genome sequences is the generally acknowledge technique of qPCR. However, rDNA is a problematic object for the quantification using qPCR for several reasons, which are discussed in detail in our previous publications (Korzeneva et al., 2016; Chestkov et al., 2018). Briefly, the qPCR efficiency is affected by the tandem nature of rDNA repeats and the presence of self-complementary regions within the repeat unit, the heterogeneity of rDNA copies with regard to methylation. In addition, qPCR efficiency is affected by the high occurrence of Gn motifs, which have the lowest oxidation potential, the existence of breaks in rDNA even in intact cells, and the reduced rDNA repair level. The situation worsens in case of quantification of rDNA in certainly damaged DNA samples (cell-free DNA, DNA of the old cells). The dependence of rDNA CN determined with qPCR on the DNA oxidation damage degree is non-linear. When the oxidation degree is low, rDNA content can be overestimated, while in case of high damage degree, it is substantially underestimated. We concluded that non-radioactive quantitative hybridization is more suitable for rDNA quantification than qPCR. This method is practically independent of DNA damage (Chestkov et al., 2018).

Unlike TR, the rDNA CN determined by the hybridization method does not change under stress conditions (Konkova et al., 2020; Umriukhin et al., 2022) and is the same in various brain structures (Ershova et al., 2019). We believe that rDNA CN is a stable genetic trait that does not change during normal aging (except for the possible loss of hypermethylated copies). However, this interesting and controversial issue requires further study.

In recent years, we have received more and more evidence that rDNA CN can be a marker allowing to predict a person’s life expectancy. At first glance, it seems paradoxical that people with a high rDNA CN, potentially increasing the ability of cells to respond to stress, do not live to old age (Malinovskaya et al., 2018 and Figure 1). However, the association of such life-shortening diseases as schizophrenia and cystic fibrosis with a large rDNA CN in the genome (Chestkov et al., 2018; Kondratyeva et al., 2021) suggests that people who are born with a large rDNA CN contain negative mutations in the genome. The development of such an embryo requires more intense protein synthesis during embryonic development. Otherwise, such embryos do not survive. Large rDNA amount helped these embryos survive, but the mutations present in the genome shorten human life expectancy. However, very low rDNA level in the cells of the body also does not contribute to longevity, since it can provide a relatively low level of protein synthesis in response to stress leading to insufficient repair of cellular damage and the accumulation of mutations that accelerate aging.

**FIGURE 4:**
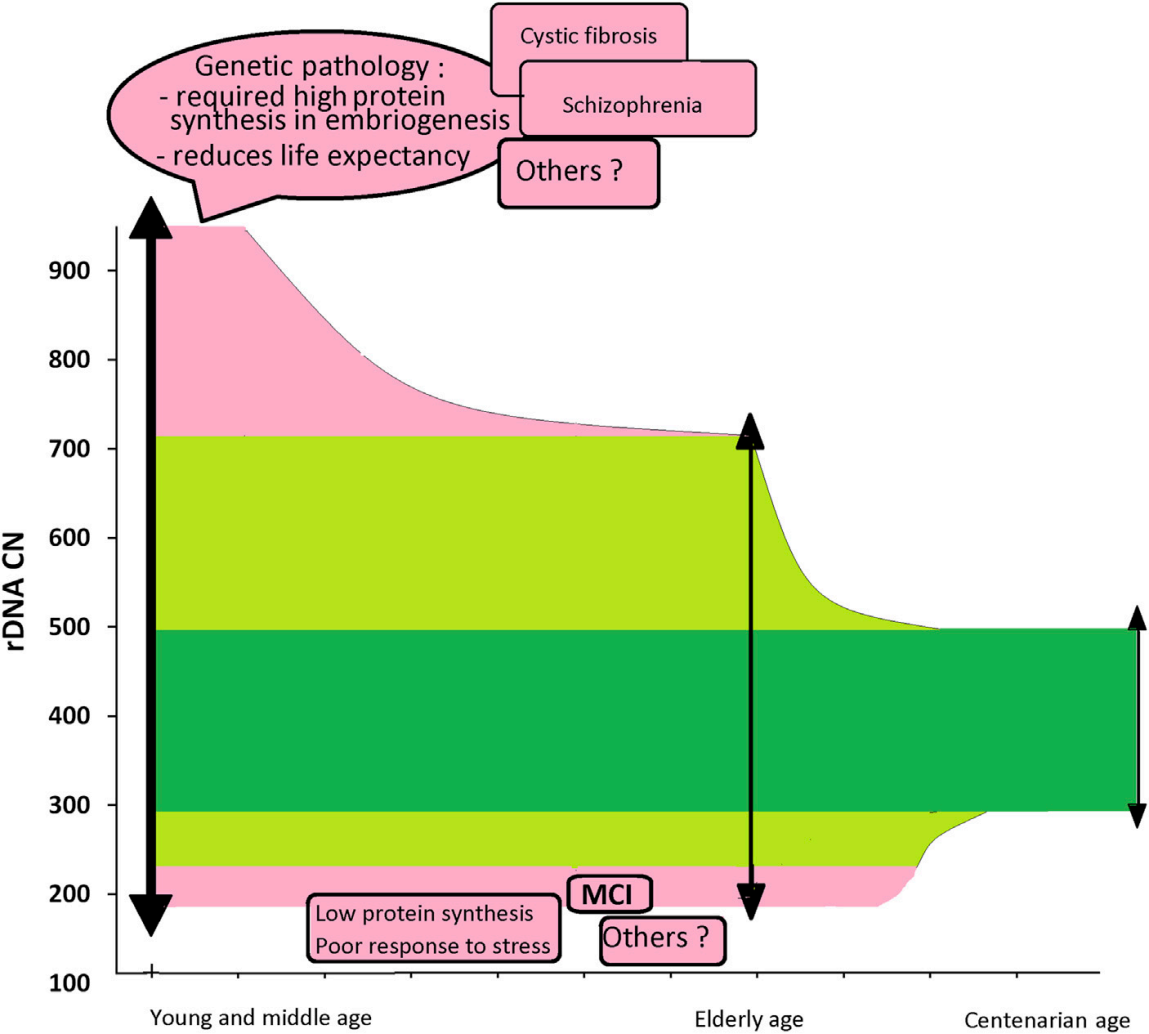
A diagram summarizing our data associating rDNA CN in the human genome with factors affecting life expectancy. A population of children: rDNA CN varies from 180–200 to 900–950. The distribution of DNA samples from the children whose genome contains mutations associated with life-shortening diseases is shifted towards high rDNA CN values. The elderly population (60–90y): rDNA CN varies from 200–220 to 500–700. The subpopulation of sick children who live to old age does not contain DNA samples with a large rDNA CN. Centenarians: rDNA CN varies in a narrow range from 300 to 500.

Figure 4 shows a diagram illustrating the hypothetical (still speculative) role of rDNA CN as a prognostic marker of life expectancy and the presence of pathology in the genome requiring high protein synthesis level. The diagram shows the facts obtained so far. In the general population of young people, including a certain number of patients with a genetic pathology shortening life expectancy, rDNA content varies in a wide range from 180–200 to 900–950 copies (Chestkov et al., 2018; Kondratyeva et al., 2021). In a subpopulation of healthy individuals, rDNA content varies in a range from 200 to 711 copies. In the group of centenarians, rDNA CN varies in a narrow range from 300 to 500 copies (Malinovskaya et al., 2018). People with low rDNA CN can potentially live more than 80 years, but they are under risk of MCI development. People with a very high rDNA CN (>700), apparently, do not live to old age probably due to a genetic pathology shortening their life expectancy. Apparently, the loss of hypermethylated copies in the same individuals with aging also contributes to the narrowing of the rDNA interval in centenarians. However, this contribution is not significant, since only 10% of people contain hypermethylated copies (Lyapunova and Veiko, 2010).

TR content on rDNA CN dependence (Figure 3) also speaks in favor of the presented scheme. It is known that genetic pathology leading to a life expectancy reduction is associated with a telomere length decrease (Codd et al., 2021). In the genomes of people belonging to the HC group with rDNA CN > 540, the TR content varies in a narrower range compared to genomes that contain low and medium rDNA amounts. In this subgroup, there are no genomes with a TR content of more than 450 pg/µg DNA (corresponding to an average telomere length of 9.1 kb), which indicates the presence of factors shortening the telomere length. On the other hand, in the HC subgroup (rDNA CN > 540) there are no genomes with a very low TR content (below 220 pg/µg DNA), which may indicate a positive role of increased rDNA CN in response to aging-associated oxidative stress, leading to telomere length reduction.

Apparently, further studies of the association of rDNA CN with pathology and the duration and quality of human life in old age will allow us to expand the scheme (Figure 4) in order to apply this indicator of rDNA CN in diagnostic medicine and gerontology.

## Data Availability

The original contributions presented in the study are included in the article/supplementary material, further inquiries can be directed to the corresponding author.
